# Phase-controlled Fourier-transform spectroscopy

**DOI:** 10.1038/s41467-018-06956-x

**Published:** 2018-10-25

**Authors:** Kazuki Hashimoto, Takuro Ideguchi

**Affiliations:** 1 Department of Physics, The University of Tokyo, Tokyo, 113-0033 Japan; 2 Aeronautical Technology Directorate, Japan Aerospace Exploration Agency, Tokyo, 181-0015 Japan; 3 PRESTO, Japan Science and Technology Agency, Tokyo, 113-0033 Japan

## Abstract

Fourier-transform spectroscopy (FTS) has been widely used as a standard analytical technique over the past half-century. FTS is an autocorrelation-based technique that is compatible with both temporally coherent and incoherent light sources, and functions as an active or passive spectrometer. However, it has been mostly used for static measurements due to the low scan rate imposed by technological restrictions. This has impeded its application to continuous rapid measurements, which would be of significant interest for a variety of fields, especially when monitoring of non-repeating or transient complex dynamics is desirable. Here, we demonstrate highly efficient FTS operating at a high spectral acquisition rate with a simple delay line based on a dynamic phase-control technique. The independent adjustability of phase and group delays allows us to achieve the Nyquist-limited spectral acquisition rate over 10,000 spectra per second, while maintaining a large spectral bandwidth and high resolution. We also demonstrate passive spectroscopy with an incoherent light source.

## Abstract (web summary)

Fourier transform spectrometers are generally limited to slow scanning rates at high resolution. Here the authors demonstrate highly efficient Fourier transform spectroscopy using a dynamic phase-control technique that enables fast acquisition without compromising bandwidth or resolution.

## Introduction

Fourier-transform spectroscopy (FTS) has provided solutions to countless chemical analysis problems in the fields of chemistry, pharmacy, medicine, biology, and physics including material science^1–3^. It enables the simultaneous identification of multiple molecular species via high-resolution broadband molecular spectra measured by a simple optical system consisting of a scanning Michelson interferometer and a broadband light source. The fact that this analytical method has long been widely used owes to the simple and robust autocorrelation-based working principle, enabling a variety of uses. For example, since it allows us to measure temporally incoherent light, FTS can be used as a passive spectroscopy technique with an external light source such as sunlight^4,5^. However, FTS has been mostly used for measuring static samples because of the low temporal resolution due to the low scan rate of mechanical delay lines. This is incompatible for investigating fast phenomena at a higher temporal resolution, such as combustion processes which often require a measurement rate over 10 kHz^6,7^. Rapid-scan FTS instruments typically operate at an improved temporal resolution only at a low spectral resolution, for example a scan rate of 1 kHz with a resolution of 120 GHz (4 cm^−1^)^8^ or 77 kHz with a resolution of 360 GHz (12 cm^−1^)^9^. For a higher resolution applicable to gas-phase analysis such as 15 GHz (0.5 cm^−1^), the available scan rate significantly drops down to less than 10 Hz^10,11^ due to the lack of a rapid-scan long-range delay line. Step-scan FTS^12^ is also known to improve the temporal resolution but it works only for measuring repeatable phenomena, which significantly limits applications. Another type of broadband absorption spectroscopy technique, called time-resolved frequency comb spectroscopy (TRFCS)^13,14^, has been used for analyzing transient chemical phenomena of gaseous molecules with high spectral resolution. The scan rate of this technique is 250 Hz, which is limited by the frame rate of the image sensor. The temporal resolution can be as high as 25 μs through repetitive measurements, but it also works only for measuring repeatable phenomena.

A recent development in dual-comb spectroscopy (DCS)^15,16^, a mechanical-scan-free FTS technique based on mutually coherent laser frequency combs with slightly detuned pulse repetition rates, has elegantly solved the problem and shown a tremendous improvement on the measurement scan rate^17–34^. The superiority of this technique is its highest scan rate, i.e., Nyquist-limited scan rate, enabled by fully utilizing the Nyquist range, determined by half a repetition rate of the laser. In other words, DCS has been a unique FTS that enables the highest measurement rate, spectral bandwidth and resolution to be achieved under the Nyquist-limited trade-off constraint. This capability is owed to the adjustable difference in both the repetition rate and carrier-envelope offset (CEO) frequency between the combs. The former determines the down-conversion factor from the optical to radio frequency (RF) to achieve the highest possible scan rate, while the latter adjusts the position of the RF spectrum within the Nyquist range for avoiding aliasing. The latter capability has not often been clearly appreciated in the previous reports on DCS, but we emphasize that it assures DCS to be an efficient FTS technique. In return for this superior capability, DCS requires a more elaborate and sophisticated system than the conventional Michelson-type FTS because DCS is based on the cross-correlation measurement between the two lasers, for which mutual coherence is usually fragile. Furthermore, it is an active-type spectrometer, which requires light sources in the instrument, and is not suitable to be used in a passive manner^20,22^.

In this work, we revisit conventional Michelson-type FTS and develop a Nyquist-limited highly efficient FTS, called phase-controlled FTS (PC-FTS), that allows us to arbitrarily adjust the scan rate, spectral bandwidth, and spectral resolution under a tradeoff relation among them. This approach uses a rapid-scan phase-control mechanism inspired by pulse shaping techniques^35^, that enables us to acquire interferograms continuously at a rate beyond 10 kHz. This is the highest scan rate achievable as a Michelson-type FTS, while avoiding spurious spectral distortions due to the aliasing effect. This is made possible by the arbitrary and independent adjustment of the group and phase delay added by the delay line, which can be understood as an analogy of the independent adjustment of the difference in repetition rate and CEO frequency in a dual-comb spectrometer. As a proof of concept demonstration, we measure broadband gas absorption spectra of hydrogen cyanide (H^12^C^14^N) and acetylene (^12^C_2_H_2_) in the near-infrared region spanning over 1.8 THz with a resolution of 11.5 GHz at an acquisition rate of over 12 kHz both with temporally coherent and incoherent light sources. Our system holds great promise for applications aimed at measuring non-repeating phenomena at high-temporal-resolution such as a transient combustion process, or a large number of events at high-throughput such as large-area environmental monitoring.

## Results

### Principle of PC-FTS

**Fig. 1:**
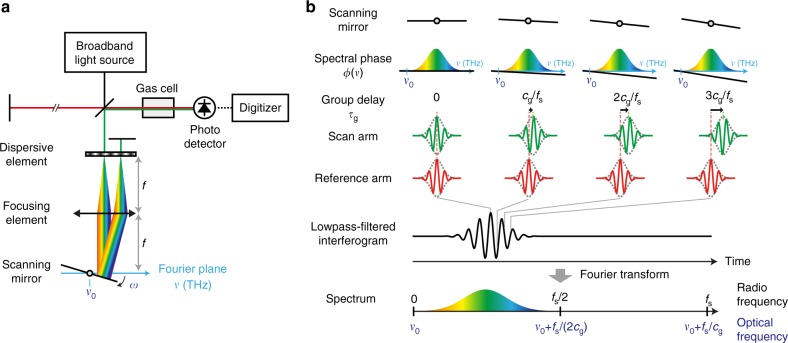
Schematic and conceptual illustration of phase-controlled Fourier-transform spectrometer. **a** Schematic of the system. The system consists of a broadband light source, a scanning Michelson interferometer with the rapid-scan phase-controlled delay line, a sample gas cell, a photodetector, and a digitizer. The delay line consists of a dispersive element, a focusing element, and a scanning mirror aligned in a reflective 4f-configulation. The broadband light is focused at the Fourier plane in the 4f system after spectral separation by the dispersive element such that each spectral component is mapped on a different position at the Fourier plane. The scanning mirror changes its angle at an angular frequency of *ω* and reflects the light with angled directions. The beam traveling through the 4f system is retro-reflected with an end mirror and goes back along the same path. The corresponding optical frequency of the pivot position of the scanning mirror in the Fourier plane is indicated as *ν*_0_. **b** Concept of PC-FTS. The upper part shows how the interferogram is obtained in the time domain. For simplicity, the light fields are depicted as a pulse train with zero carrier-envelope-phase as shown in the pulses in the reference arm. Here, *f*_s_ denotes the sampling rate, which may be determined by the pulse repetition rate or sampling rate of the digitizer. At each time frame, the light field in the scan arm acquires a linear spectral phase that is proportional to the angle of the scanning mirror in the delay line. The linear spectral phase ramp adds a group delay and phase delay to the light field. The pivot frequency *ν*_0_ is converted to the zero frequency in the down-converted RF spectrum after Fourier-transformation. Therefore, the down-converted spectrum can be positioned inside of the Nyquist range (0−*f*_s_/2) by adjusting *ν*_0_

**Fig. 2:**
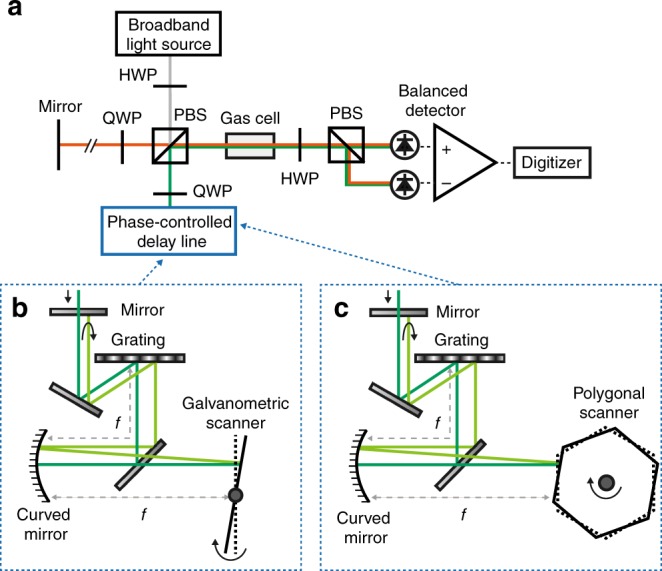
Detailed schematic of the phase-controlled FTS. **a** Detailed schematic of PC-FTS. HWP Half-wave plate, QWP Quarter-wave plate, PBS Polarizing beamsplitter. **b** Phase-controlled delay line with a galvanometric scanner. **c** Phase-controlled delay line with a polygonal scanner

A schematic of PC-FTS is shown in Fig. 1a. It consists of a broadband light source, a scanning Michelson interferometer, a single photodetector and a digitizer. Since Michelson-type FTS is an autocorrelation-based measurement, there is no requirement on the temporal coherence for the measured light source. In our experiment, a coherent mode-locked laser or an incoherent amplified spontaneous emission from a super-luminescent diode (SLD) is used as a broadband light source. The scanning Michelson interferometer varies a time delay between the beams traveling along the reference and scan arms, so that an autocorrelation trace, called the interferogram, is obtained as a function of time by the photodetector. The digitized interferogram is Fourier-transformed to obtain a spectrum. By placing a sample in the optical path between the interferometer and the detector, its absorption feature can be encoded onto the spectrum. Our phase-controlled delay line consists of a dispersive element such as a diffraction grating, a focusing element and a scanning mirror, which are aligned in a 4f geometry such that each spectral component of the beam is focused at a different spot on the Fourier plane, where the scanning mirror is placed^36,37^. The reflected beam from the scanning mirror then passes through the optical elements in the 4f system and is retro-reflected by an end mirror. The 4f geometry ensures the beam travels back along the incident path for any facet-angles of the scanning mirror. Using a rapidly scanning mirror enables the system to function as a rapid-scan FTS. A detailed schematic of the PC-FTS is shown in Fig. 2 and the components used in the system are described in Methods section.

The working principle of PC-FTS is depicted in Fig. 1b. A slight inclination of the scanning mirror placed at the Fourier plane adds a linear spectral phase ramp to the broadband light that travels through the delay line. Here, we describe the system with a galvanometric scanner. Assuming the scanning mirror rotates clockwise at a constant angular frequency *ω*, the added spectral phase *ϕ*(*ν*) and group delay *τ*_g_ may be described as:

1 $$\phi \left( \nu \right) \approx - 2{\mathrm{\pi }}c_{\mathrm{g}}\left( {\nu - \nu _0} \right)t$$

2 $$\tau _{\mathrm{g}} = - \frac{1}{{2{\mathrm{\pi }}}}\frac{{\partial \phi \left( \nu \right)}}{{\partial \nu }} \approx c_{\mathrm{g}}t$$

where *t* denotes time, *ν* optical frequency, *ν*_0_ the optical frequency corresponding to the pivot position of the scanning mirror in the Fourier plane, and *c*_g_ ∝ *ω*/*ν*_0_ the down-conversion factor, which is approximately a constant value in time. A full description of *c*_g_ is described in Supplementary Note 1. The group delay is linearly proportional to time by a factor of *c*_g_, while the group velocity can be described as *c*_g_*c*, where *c* denotes the speed of light. This indicates that a high angular frequency of the scanning mirror makes possible a large bandwidth of the down-converted RF spectrum, leading to an efficient use of the Nyquist range. Adjusting the angular frequency of the scanning mirror, which determines the down-conversion factor of FTS, is analogous to adjusting the difference in repetition rate between the combs in DCS. Equation 1 tells us that a down-converted RF spectrum has a frequency of *c*_g_(*ν* − *ν*_0_), that can be shifted by changing the pivot position of the scanning mirror *ν*_0_. Adjusting *ν*_0_ allows us to fully utilize the Nyquist range while avoiding the aliasing, which is analogous function as that of the difference in CEO frequency between the combs in DCS. The Nyquist theorem tells us the trade-off relation among the scan rate *f*_scan_, spectral bandwidth Δ*ν*, and spectral resolution *δν*, which may be described as:

3 $$f_{{\mathrm{scan}}}{\mathrm{\Delta }}\nu \frac{1}{{\delta \nu }} < \frac{{f_{\mathrm{s}}}}{2},$$

where *f*_s_ denotes the sampling rate. The detailed description of Inequality 3 is provided in Supplementary Note 2.

### Phase and group delay correction

Since the down-conversion factor *c*_g_ is not exactly a constant value in time, the phase-controlled delay lines generate temporally nonlinear phase and group delays, which must be corrected for FTS. The detailed description of the nonlinearity is provided in Supplementary Note 1. To linearize both the nonlinear phase and group delays, we use continuous-wave (CW) interferograms at two different optical frequencies and follow the correction procedure that is in principle the same as that of DCS^19,38^. Note that the fixed instrumental nonlinearities of the phase and group delays are corrected in the PC-FTS, while the random temporal fluctuations of the difference in repetition rate and CEO frequency are corrected in the DCS. Since the PC-FTS is an autocorrelation-based technique where random temporal fluctuation is inherently small, the correction interferograms are not necessarily measured for every measurement.

**Fig. 3:**
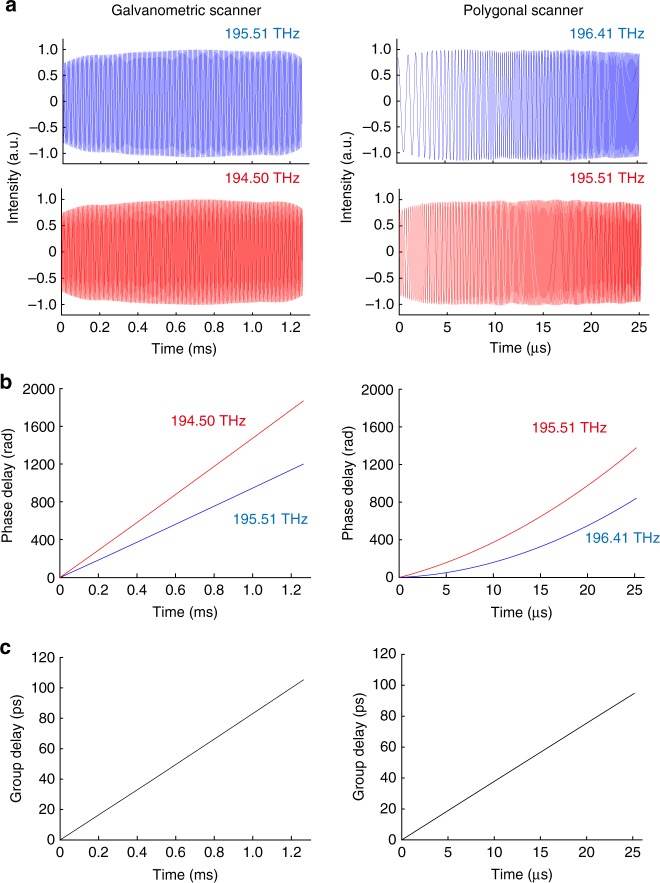
Characterization of the phase and group delays added by the delay lines. **a** Measured continuous-wave interferograms at the two different frequencies. **b** Phase delays of the CW interferograms. **c** Group delays evaluated from the phase delays at the two different frequencies

We measure the CW interferograms at two different frequencies with external-cavity laser diodes with a linewidth of less than 500 kHz lasing at 195.51 and 194.50 THz for the system with a galvanometric scanner, and 196.41 and 195.51 THz for the system with a polygonal scanner. The lasers’ frequencies are measured by an optical spectrum analyzer (AQ6317B, Yokogawa) with the accuracy of ±3 GHz. The measured CW interferograms are shown in Fig. 3a. The phase nonlinearity is seen especially in the data measured by the system with the polygonal scanner. The phase delays are extracted from the interferograms and plotted in Fig. 3b. The group delays are calculated from the phase delays at the two different frequencies as shown in Fig. 3c. The maximum group delay is about 100 ps, which corresponds to the spectral resolution of about 10 GHz.

### Broadband absorption spectroscopy

**Fig. 4:**
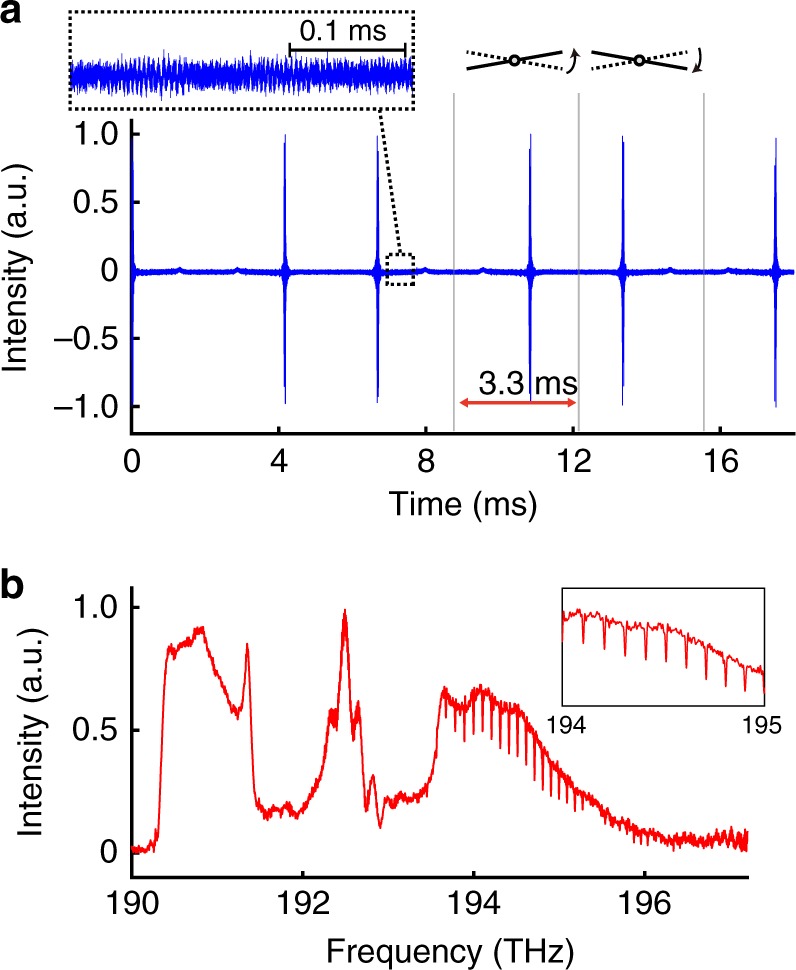
Broadband spectroscopic measurement by PC-FTS based on a galvanometric scanner. **a** Continuous interferograms of H^12^C^14^N molecules measured with the mode-locked laser. The single-sided interferograms are continuously obtained at a scan rate of 300 Hz (the corresponding temporal interval is 3.3 ms). The inset shows modulations due to the free-induction decay. **b** A broadband spectrum obtained by Fourier-transforming a single interferogram. The non-averaged broadband spectrum covering 7 THz shows absorption lines of HCN molecules at a spectral resolution of 10.1 GHz with a high signal-to-noise ratio. The structured spectral profile comes from that of the mode-locked fiber laser

As a proof-of-concept demonstration of the PC-FTS, we measure broadband absorption spectra of gaseous HCN by the system with a galvanometric scanner. An Er-fiber mode-locked laser that generates femtosecond pulses at a repetition rate of 50 MHz is used as a light source. A full description of the system is provided in Methods section. The interferograms shown in Fig. 4a display the clear modulations of the molecular free-induction decay. By Fourier-transforming a single-sided interferogram with the aforementioned phase and group delay correction^19,38^ and the conventional phase correction methods^39–43^, a broadband spectrum spanning over 7 THz is obtained at a resolution of 10.1 GHz with a high signal-to-noise ratio (SNR) (Fig. 4b). Sharp absorption lines of the overtone vibrational bands of HCN are clearly observed. Note that the resolution of 10 GHz corresponds to the maximum optical path length difference of 30 mm in a conventional FTS, which is achieved only by tilting the scanning mirror in the PC-FTS. The scan rate is 300 Hz, which is not limited by the Nyquist range (0–25 MHz) determined by the pulse repetition rate of the mode-locked laser but the scan rate of the galvanometric scanner.

### Nyquist-limited rapid-scan spectroscopy

**Fig. 5:**
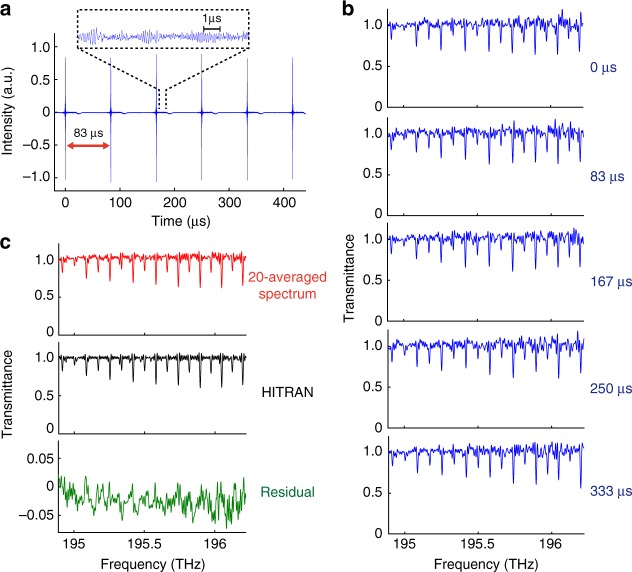
High-scan-rate broadband spectroscopic measurement by PC-FTS based on a polygonal scanner. **a** Continuous interferograms of ^12^C_2_H_2_ molecules measured with the mode-locked laser. The burst of the interferogram appears every 83 µs, corresponding to a scan rate of 12 kHz. Clear modulations of the molecular free-induction decay are observed. **b** Non-averaged transmission spectra corresponding to the interferograms shown in Fig. 5a. Each spectrum covers over 1.5 THz with a resolution of 11.5 GHz. The absorption lines of C_2_H_2_ are clearly observed. The noise in the spectra can mainly be attributed to the side-modulations of the sinc function caused by the rectangular Fourier-transform windowing. Apodization (not applied here) can reduce the side-modulations at the expense of the spectral resolution. **c** Comparison between a 20-averaged transmission spectrum and a computationally calculated spectrum based on HITRAN database. The standard deviation of the residual is 1.7%

To fully utilize the Nyquist range and achieve the Nyquist-limited highest scan rate, a polygonal scanner can be used instead of the galvanometric scanner. With the faster rotation speed of the polygonal scanner, a continuous measurement of interferograms at a rate of 12 kHz is demonstrated in Fig. 5a. Figure 5b shows the corresponding consecutive broadband C_2_H_2_ absorption spectra with a bandwidth of 1.5 THz at a spectral resolution of 11.5 GHz. The spectra are normalized and converted to transmittance by a baseline fitting with Savitzky–Golay filtering^44^. Note that the effective scan velocity calculated by (scan rate) × (maximum group delay) × *c* is 340 m s^−1^, which is orders of magnitude higher than those of conventional delay lines. The down-conversion factor, (effective scan velocity)/*c* = (scan rate) × (maximum group delay), is about 10^−6^. Figure 5c shows a 20-averaged spectrum compared to a computationally retrieved spectrum based on HITRAN database^45^. The SNR of a single spectrum is 54, which is limited by detector noise in this proof-of-concept demonstration. It could be improved by using a detector with better noise performance. The detailed discussion on SNR is provided in Supplementary Note 3.

### PC-FTS with an incoherent light source

Since the PC-FTS is an autocorrelation-based technique, it functions as a passive spectrometer with a temporally incoherent light source. Furthermore, since a repetition rate is not usually defined for an incoherent light, the Nyquist frequency of the measurement is determined by an electronic sampling rate of a digitizer. This enables a higher scan rate with a larger Nyquist range.

**Fig. 6:**
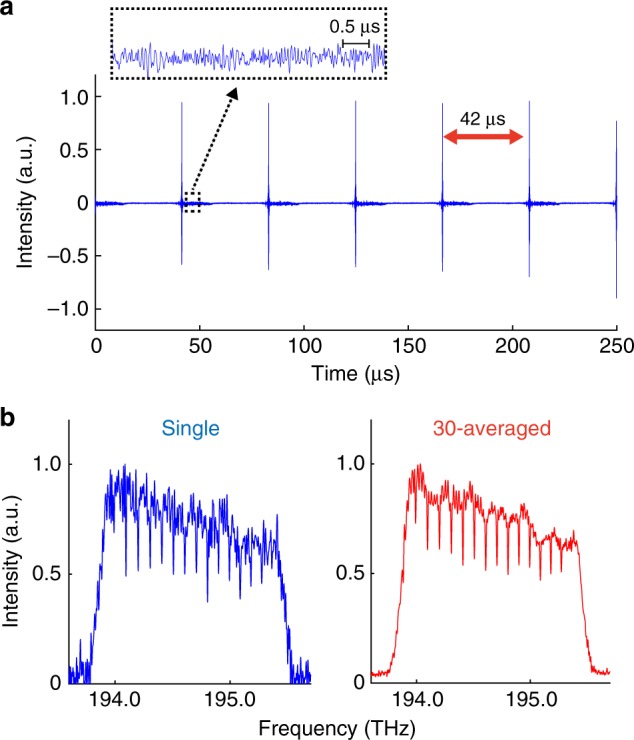
Interferograms and spectra measured by the PC-FTS with the SLD. **a** Continuous interferograms of H^12^C^14^N molecules measured with the SLD at a scan rate of 24 kHz. **b** Spectra obtained by Fourier-transforming a single interferogram (left), and a 30-averaged spectrum

To show the applicability of an incoherent light to the PC-FTS, we replace the mode-locked laser by a commercially available SLD and perform a proof-of-concept measurement. We acquire HCN absorption spectra over 1.8 THz with a spectral resolution of 11.5 GHz at a scan rate of 24 kHz, which is higher than that of the mode-locked laser by a factor of two. Note that the scan rate of 24 kHz is not achievable with the mode-locked laser at the repetition rate of 50 MHz because of the aliasing effect caused by the Nyquist constraint due to the pulse repetition rate. Measured continuous interferograms are shown in Fig. 6a, where free-induction decay of the molecules is clearly seen every 42 μs. A spectrum Fourier-transformed from a single interferogram and a 30-averaged spectrum are shown in Fig. 6b. The fringe observed in each spectrum is the ripple-noise due to the multiple-reflections between the facets of the SLD chip.

## Discussion

To see how efficiently the PC-FTS uses the Nyquist range, it is helpful to estimate the bandwidth of the down-converted spectrum when measuring with a conventional FTS. Assuming a commercially available FTS system that can measure a broadband near-infrared spectrum spanning from 176 to 333 THz with a spectral resolution of 7.5 GHz at a scan rate of 1.9 Hz, the down-converted spectrum appears at 45–84 kHz, which is much narrower than the Nyquist range set by the sampling rate of a few tens of MHz. The down-conversion factor is 2.5 × 10^−10^, which is orders of magnitude smaller than that of the PC-FTS (10^−6^).

Comparing the two Nyquist-limited FTS (DCS and PC-FTS) is useful to see the characteristics of these methods. DCS is a mechanical-scan-free FTS, therefore the system can be robust. Additionally, this frequency comb based FTS can assure high frequency accuracy. On the other hand, PC-FTS works with a single light source, which can be an incoherent light source, making the system simple. Also, this autocorrelation-based FTS can be used as a passive spectrometer.

The concept of this technique can be applied for other wavelengths including the mid-infrared region, where the fundamental vibrational modes of molecules exist. The single-photodetector-operation of the FTS is especially advantageous in the mid-infrared region because of the lack of a high-quality mid-infrared detector-array required for a high-speed dispersive spectrometer. Furthermore, PC-FTS is useful not only for gas-phase molecules but also for liquid-phase or solid-phase materials. As discussed above, since we can arbitrarily choose a combination of the scan rate, spectral bandwidth and resolution, a broader spectral bandwidth, for example, can be measured by reducing the spectral resolution while keeping the scan rate. Finally, this highly efficient FTS technique can also be modified and applied to multi-dimensional FTS, which requires time-consuming scans of multiple delay lines^32^.

## Methods

### Phase-controlled delay line

Our phase-controlled delay line consists of a dispersive element, a focusing element, a scanning mirror, and a flat mirror. These components are aligned to form a reflective 4f configuration such that a spectrum of the incident light is focused onto the Fourier plane where the scanning mirror is placed. In our demonstration, a reflective ruled grating with 600 lines mm^−1^ and a curved mirror with a focal length of 150 mm are used as a dispersive element and a focusing element, respectively. The number of grooves of the grating and the focal length of the curved mirror are to be set for optimizing the system (See Supplementary Note 1 for details). A galvanometric scanner or a polygonal scanner with 36 facets are used as the scanning mirror. The mirror facets of both scanners are coated with gold. The facet size of the galvanometric scanner is 10 mm, and that of the polygonal scanner is 5.6 mm per each facet. The inner diameter of the polygonal scanner is 63.6 mm and its rotation speed is set to be 20,000 or 40,000 rpm (rotations per minute) in our experiments. Replacing these optical components to ones with different parameters allows us to choose different combinations of the scan rate, spectral bandwidth and resolution.

### Phase-controlled FTS

A full description of our PC-FTS is shown in Fig. 2. A temporally coherent mode-locked Er-doped fiber laser (Femtolite CS-20-GS, IMRA) or an incoherent SLD (S5FC1005S, Thorlabs) is used as a broadband light source. The broadband light is delivered with a single-mode fiber to a fiber-collimator placed at the input port of the spectrometer. For a rapid measurement with the polygonal scanner, the light is optically band-pass-filtered with a span of 14 nm (corresponding to 1.8 THz) centered at 1533.5 nm (FBH1550-12, Thorlabs). The collimated light is split into two beams with a half-wave plate and a polarizing beamsplitter. The rotation angle of the half-wave plate is set to have a power ratio of 50:50 between the two output beams of the interferometer. While the beam in the reference arm is retro-reflected by a flat mirror, the other beam in the scan arm travels through the phase-controlled delay line and retro-reflected with a time delay. A quarter-wave plate inserted in each arm rotates the polarization of the reflected beam by 90 degrees, so that the recombined beams exit from the output port of the interferometer with orthogonal polarizations. The beams go through a gas cell containing either H^12^C^14^N (TT-HCN-1OOT-G-Q, triad technology) or ^12^C_2_H_2_ (TT-CH12-50T-G-Q, triad technology) in a two-way (round trip) geometry and detected by an InGaAs balanced photodetector (PDB415C-AC, Thorlabs). For balancing the photodiodes’ signals, a half-wave plate and a polarizing beamsplitter are used in front of the detector. The average power of the detected light is set to be 15 µW for each photodiode to avoid effects of detector nonlinearity. The detector signal is lowpass-filtered at 21 MHz when using the mode-locked laser. The signal is digitized by a data acquisition board (ATS9440, AlazarTech) at a sampling rate of 125 MS s^−1^. The digitized time domain data is segmented into independent interferograms and each of them is Fourier-transformed after the phase correction.

### Code availability

The Matlab code used for analyzing the data of this study are available from the corresponding author upon reasonable request.

## Electronic supplementary material

Supplementary Information

## Data Availability

The data that support the findings of this study are available from the corresponding author upon reasonable request.
