# Supplementary material for: Phase-controlled Fourier-transform spectroscopy
Source: Nat Commun. 2018 Oct 25;9:4448. doi: 10.1038/s41467-018-06956-x (PMC6202327; doi:10.1038/s41467-018-06956-x)
Supplement: Supplementary file 1 — Supplementary Information [file 41467_2018_6956_MOESM1_ESM.pdf]

Supplementary Information: Phase-controlled Fourier-transform spectroscopy

Kazuki Hashimoto[1,2], Takuro Ideguchi[1,3*]

[1]Department of Physics, The University of Tokyo, Tokyo 113-0033, Japan
[2]Aeronautical Technology Directorate, Japan Aerospace Exploration Agency, Tokyo 181-0015, Japan
[3]PRESTO, Japan Science and Technology Agency, Tokyo 113-0033, Japan
*ideguchi@phys.s.u-tokyo.ac.jp

Supplementary Note 1: Theoretical description of the phase-controlled FTS

Phase-controlled delay line with a galvanometric scanner

Theoretical description of the phase-controlled delay line is provided as follows. The detailed schematic of the phase-controlled delay line with a galvanometric scanner is depicted in Supplementary Figure 1**a**. In this configuration, the grating equation may be described as:

$$\sin\beta_{\lambda} = N\lambda - \sin\alpha, \tag{1}$$

$$\sin\beta_{\lambda_0} = N\lambda_0 - \sin\alpha, \tag{2}$$

where β_λ and β_{λ_0} denote the first-order diffraction angles of the light at wavelength of λ and λ_0, α incident angle, N groove density of the grating. Here, λ_0 denotes the wavelength corresponding to the pivot position of the scanning mirror in the Fourier plane. Assuming the facet of the scanning mirror is on the Fourier plane at $t = 0$, an optical path length difference $L_\mathrm{g}(t)$ between the beams at the wavelength of λ and λ_0 is described as:

$$L_\mathrm{g}(t) = l_\mathrm{f}\left(\tan\beta_{\lambda_0} - \tan\beta_{\lambda}\right)\tan\omega t, \tag{3}$$

where l_f denotes the focal length of the curved mirror and ω the angular frequency of the scanning mirror. When $\beta_{\lambda_0}, \beta_\lambda \ll 1$ and $\omega t \ll 1$, $L_\mathrm{g}(t)$ is described as:

$$\begin{aligned} L_\mathrm{g}(t) &\approx l_\mathrm{f}N(\lambda_0 - \lambda)\omega t \\ &\approx l_\mathrm{f}Nc\left(\frac{\nu - \nu_0}{\nu_0\nu}\right)\omega t, \end{aligned} \tag{4}$$

where c is the speed of light, ν and ν_0 the optical frequency corresponding to λ and λ_0. Then, the spectral phase $\phi_\mathrm{g}(\nu)$ may be described as:

$$\begin{aligned} \phi_\mathrm{g}(\nu) &= -\frac{2\pi\nu}{c} \times 4 \times L_\mathrm{g}(t) \\ &\approx -2\pi\,\frac{4l_\mathrm{f}N\omega}{\nu_0}(\nu - \nu_0)t \\ &= -2\pi\,c_\mathrm{g}(\nu - \nu_0)t, \end{aligned} \tag{5}$$

where $c_\mathrm{g} = \frac{4l_\mathrm{f}N\omega}{\nu_0}$ is the down conversion factor. Since the light is reflected off the scanning mirror twice in the delay line, the total optical path length difference is $L_\mathrm{g}(t)$ multiplied by 4. The group delay τ_g may be described as:

$$\tau_{\mathrm{g}} = -\frac{1}{2\pi}\frac{\partial \phi_{\mathrm{g}}(\nu)}{\partial \nu} = c_{\mathrm{g}} t. \tag{6}$$

Phase-controlled FTS with a galvanometric scanner

We denote complex electric fields of an input and two outputs of the Michelson interferometer as $E(t)$, $E_{\mathrm{scan}}(t)$ and $E_{\mathrm{ref}}(t)$, and their Fourier-transformed fields as $E(\nu)$, $E_{\mathrm{scan}}(\nu)$ and $E_{\mathrm{ref}}(\nu)$, respectively. In the frequency domain, $E_{\mathrm{ref}}(\nu)$ and $E_{\mathrm{scan}}(\nu)$ may be described by adding the spectral phase to $E(\nu)$ as:

$$E_{\mathrm{ref}}(\nu) = E(\nu)\exp(-\mathrm{i}2\pi\nu\tau), \tag{7}$$

$$\begin{aligned} E_{\mathrm{scan}}(\nu) &= E(\nu)\exp\left[\mathrm{i}\left\{-2\pi\nu\tau + \phi_{\mathrm{g}}(\nu)\right\}\right] \\ &= E(\nu)\exp\left[-\mathrm{i}2\pi\left\{\nu\tau + (\nu-\nu_0)\tau_{\mathrm{g}}\right\}\right], \end{aligned} \tag{8}$$

where τ denotes a delay added by the reference and scan arm of the interferometer, and τ_{g} the group delay added by the delay line in the scan arm. By inverse-Fourier-transforming $E_{\mathrm{ref}}(\nu)$ and $E_{\mathrm{scan}}(\nu)$, the complex electric fields $E_{\mathrm{ref}}(t)$ and $E_{\mathrm{scan}}(t)$ are described as:

$$\begin{aligned} E_{\mathrm{ref}}(t) &= \mathcal{F}^{-1}\{E_{\mathrm{ref}}(\nu)\} \\ &= \int_{-\infty}^{\infty} E(\nu)\exp\{\mathrm{i}2\pi\nu(t-\tau)\}d\nu \\ &= E(t-\tau), \end{aligned} \tag{9}$$

$$\begin{aligned} E_{\mathrm{scan}}(t) &= \mathcal{F}^{-1}\{E_{\mathrm{scan}}(\nu)\} \\ &= \exp\left(\mathrm{i}2\pi\nu_0\tau_{\mathrm{g}}\right)\int_{-\infty}^{\infty} E(\nu)\exp\left\{\mathrm{i}2\pi\nu\left(t-\tau-\tau_{\mathrm{g}}\right)\right\}d\nu \\ &= \exp\left(\mathrm{i}2\pi\nu_0\tau_{\mathrm{g}}\right)E\left(t-\tau-\tau_{\mathrm{g}}\right). \end{aligned} \tag{10}$$

Integrated intensity of the combined electric fields of $E_{\mathrm{ref}}(t)$ and $E_{\mathrm{scan}}(t)$ detected by a photodetector can be written as:

$$\begin{aligned} I\left(\tau_{\mathrm{g}}\right) &= \int_{-\infty}^{\infty} |E_{\mathrm{ref}}(t) + E_{\mathrm{scan}}(t)|^2 dt \\ &= \int_{-\infty}^{\infty} \left|E(t) + \exp\left(\mathrm{i}2\pi\nu_0\tau_{\mathrm{g}}\right)E\left(t-\tau_{\mathrm{g}}\right)\right|^2 dt. \end{aligned} \tag{11}$$

Here, $t-\tau$ is replaced by t for simplicity of the equation. The AC component of $I\left(\tau_{\mathrm{g}}\right)$, namely the interferogram $S\left(\tau_{\mathrm{g}}\right)$, is described as:

$$S\left(\tau_{\mathrm{g}}\right) = \exp\left(\mathrm{i}2\pi\nu_0\tau_{\mathrm{g}}\right)\int_{-\infty}^{\infty} E^*(t)E\left(t-\tau_{\mathrm{g}}\right)dt + c.c.. \tag{12}$$

Finally, Fourier-transforming the interferogram gives the spectrum:

$$\begin{aligned} \mathcal{F}\left\{S\left(\tau_{\mathrm{g}}\right)\right\} &= \int_{-\infty}^{\infty} S\left(\tau_{\mathrm{g}}\right)\exp\left(-\mathrm{i}2\pi\nu\tau_{\mathrm{g}}\right)d\tau_{\mathrm{g}} \\ &= \int_{-\infty}^{\infty} \exp\left\{-\mathrm{i}2\pi(\nu-\nu_0)\tau_{\mathrm{g}}\right\}s\left(\tau_{\mathrm{g}}\right)d\tau_{\mathrm{g}} + c.c. \\ &= B(\nu-\nu_0) + c.c.. \end{aligned} \tag{13}$$

Here, $s(\tau_{\mathrm{g}}) = \int_{-\infty}^{\infty} E^{*}(t)E(t-\tau_{\mathrm{g}})dt$ and $B(\nu) = \mathcal{F}\{s(\tau_{\mathrm{g}})\}$. As shown above, the spectrum is shifted by ν_0, which is experimentally demonstrated by changing the pivot position of the scanning mirror in the Fourier plane.

Phase-controlled FTS with a polygonal scanner

Unlike a galvanometric scanner, a polygonal scanner does not have the pivot in the Fourier plane (Supplementary Figure 1**b)**. Therefore, the delay line based on the polygonal scanner is described in a different manner. In this configuration, the optical path length difference $L_{\mathrm{p}}(t)$ added by the delay line may be described as:

$$\begin{aligned} L_{\mathrm{p}}(t) &\approx \left\{ l_{\mathrm{f}} N(\lambda_0 - \lambda) - R\tan\frac{\omega t}{2} \right\} \tan\omega t \\ &\approx l_{\mathrm{f}} N c \left(\frac{\nu - \nu_0}{\nu_0 \nu} \right) \omega t - R\frac{\omega^2 t^2}{2}, \end{aligned} \tag{14}$$

where R denotes the inner radius of the polygonal scanner that equals to the distance between the pivot and the mirror facets. The added spectral phase $\phi_{\mathrm{p}}(\nu)$ may be described as:

$$\begin{aligned} \phi_{\mathrm{p}}(\nu) &= -\frac{2\pi\nu}{c} \times 4 \times L_{\mathrm{p}}(t) \\ &\approx -2\pi \frac{4 l_{\mathrm{f}} N\omega}{\nu_0}(\nu - \nu_0)t + 2\pi \frac{2R\omega^2 \nu}{c} t^2 \\ &= -2\pi \left\{ \left(\frac{4 l_{\mathrm{f}} N\omega}{\nu_0} - \frac{2R\omega^2}{c} t \right) \nu - 4 l_{\mathrm{f}} N\omega \right\} t \\ &= -2\pi \left(\frac{4 l_{\mathrm{f}} N\omega}{\nu_0} - \frac{2R\omega^2}{c} t \right) \left(\nu - \frac{\nu_0}{1 - \frac{R\omega\nu_0}{2 c l_{\mathrm{f}} N} t} \right) t \\ &= -2\pi c_{\mathrm{p}} (\nu - \nu_0') t, \end{aligned} \tag{15}$$

where $c_{\mathrm{p}} = \left(\frac{4 l_{\mathrm{f}} N\omega}{\nu_0} - \frac{2R\omega^2}{c} t \right)$ denotes the down conversion factor and $\nu_0' = \frac{\nu_0}{1 - \frac{R\omega\nu_0}{2 c l_{\mathrm{f}} N} t}$ the optical frequency corresponding to the intersection point of the scanner's facet on the Fourier plane. Note that the above expressions become identical to those with the galvanometric scanner shown in the previous section with $R = 0$. The group delay τ_{p} may be described as:

$$\tau_{\mathrm{p}} = -\frac{1}{2\pi} \frac{\partial \phi_{\mathrm{p}}(\nu)}{\partial \nu} = c_{\mathrm{p}} t. \tag{16}$$

In the frequency domain, $E_{\mathrm{scan}}(\nu)$ may be described as:

$$E_{\mathrm{scan}}(\nu) = E(\nu)\exp\{-\mathrm{i}2\pi(\nu - \nu_0')\tau_{\mathrm{p}}\}, \tag{17}$$

Here, we omit τ for simplicity of the equation. Then, $E_{\mathrm{scan}}(t)$ is written as:

$$\begin{aligned} E_{\mathrm{scan}}(t) &= \exp(\mathrm{i}2\pi\nu_0' \tau_{\mathrm{p}}) \int_{-\infty}^{\infty} E(\nu)\exp\{\mathrm{i}2\pi\nu(t - \tau_{\mathrm{p}})\} d\nu \\ &= \exp(\mathrm{i}2\pi\nu_0' \tau_{\mathrm{p}}) E(t - \tau_{\mathrm{p}}). \end{aligned} \tag{18}$$

Therefore, the interferogram $S(\tau_{\mathrm{p}})$ is written as:

$$S(\tau_{\mathrm{p}}) = \int_{-\infty}^{\infty} E^{*}(t)E(t-\tau_{\mathrm{p}})\exp(\mathrm{i}2\,\pi\,\nu_0'\,\tau_{\mathrm{p}})dt + c.c.. \tag{19}$$

Since τ_{p} and ν_0' are temporally nonlinear variables, phase correction is necessary to retrieve the spectrum.

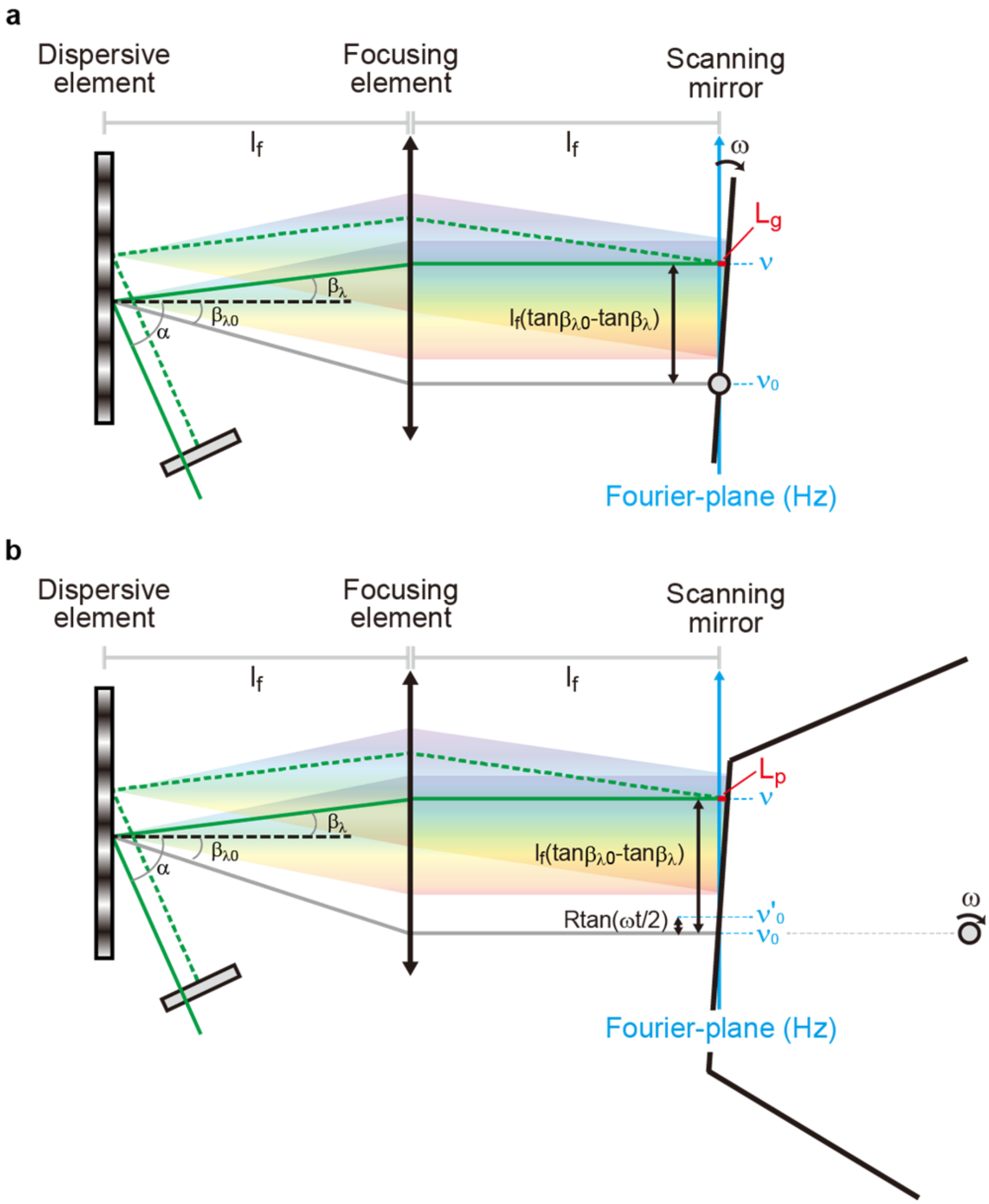

Supplementary Figure 1| Detailed schematic of the phase-controlled delay line. a, Schematic of the phase-controlled delay line with a galvanometric scanner. **b,** Schematic of the phase-controlled delay line with a polygonal scanner.

Supplementary Note 2: Trade-off relation among the scan rate, spectral bandwidth and resolution

Phase-controlled FTS with a galvanometric scanner

A trade-off relation among the scan rate, spectral bandwidth and resolution of the phase-controlled FTS with a galvanometric scanner can be derived as follows. A down-converted radio frequency $f(\nu)$ may be described as:

$$f(\nu) = \frac{4l_{\mathrm{f}}N\omega}{\nu_0}(\nu - \nu_0) = c_{\mathrm{g}}(\nu - \nu_0), \tag{20}$$

where c_{g} denotes the down conversion factor, ν_0 the optical frequency corresponding to the pivot position of the scanning mirror in the Fourier plane. All the spectral elements $f(\nu)$ of the measured light must be within the Nyquist range of the system to avoid the aliasing effect:

$$0 \leq f(\nu) < \frac{f_{\mathrm{s}}}{2}, \tag{21}$$

where f_{s} is a sampling rate of the system, which can be determined by either an optical or electrical sampling rate, namely the repetition rate of the pulsed laser or the clock rate of the digitizer, respectively. For fully utilizing the Nyquist range, we set ν_0 at the edge of the measured optical spectrum. Thus, the trade-off relation may be described with an optical bandwidth of the spectrum $\Delta\nu$ as:

$$c_{\mathrm{g}}\Delta\nu < \frac{f_{\mathrm{s}}}{2}. \tag{22}$$

The spectral resolution $\delta\nu$ determined by the maximum group delay $\tau_{\mathrm{g,max}}$ is described as:

$$\delta\nu = \frac{1}{\tau_{\mathrm{g,max}}} = \frac{f_{\mathrm{scan}}}{c_{\mathrm{g}}}, \tag{23}$$

where f_{scan} denotes the scan rate. Finally, the trade-off relation among the scan rate, spectral bandwidth and resolution can be described as:

$$f_{\mathrm{scan}}\Delta\nu\frac{1}{\delta\nu} < \frac{f_{\mathrm{s}}}{2}. \tag{24}$$

Phase-controlled FTS with a polygonal scanner

When using a polygonal scanner as a scanning mirror, the above expressions are modified as follows. The down-converted radio frequency of the spectrum may be described as:

$$f(\nu) = \frac{4l_{\mathrm{f}}N\omega}{\nu_0}(\nu - \nu_0) - \frac{2R\omega^2}{c}\nu t. \tag{25}$$

All the spectral elements $f(\nu)$ must be in the Nyquist range of the system for avoiding the aliasing effect:

$$0 \leq \frac{4l_{\mathrm{f}}N\omega}{\nu_0}(\nu - \nu_0) - \frac{2R\omega^2}{c}\nu t < \frac{f_{\mathrm{s}}}{2}. \tag{26}$$

This configuration allows us to use a part of the measurement time in order to avoid the aliasing effect. To consider the duty cycle D, we introduce t_{start} and t_{end} $(-\frac{1}{f_{\mathrm{scan}}} < t_{\mathrm{start}}, t_{\mathrm{end}} \leq 0)$ for expressing the start and end time of the single interferogram, respectively. The duty cycle may be written as:

$$D = (t_{\mathrm{end}} - t_{\mathrm{start}})f_{\mathrm{scan}}. \tag{27}$$

Assuming the spectrum spanning from ν_0 to $\nu_0 + \Delta\nu$ mapped on the Fourier plane, t_{start} and t_{end} must satisfy the following inequalities:

$$t_{\mathrm{start}} > \frac{c}{2R\omega^2}\frac{1}{(\nu_0 + \Delta\nu)}\left(\frac{4l_{\mathrm{f}}N\omega}{\nu_0}\Delta\nu - \frac{f_{\mathrm{s}}}{2}\right), \tag{28}$$

$$t_{\mathrm{end}} \leq 0. \tag{29}$$

Note that since the frequency of each spectral element decreases in time, the highest available frequency $\frac{f_{\mathrm{s}}}{2}$ determines the start time t_{start}, while the lowest frequency zero the end time t_{end}. Here, we consider a case where $t_{\mathrm{end}} = 0$ so that we have the longest duty cycle. Supplementary Inequality 28 can be described as:

$$c_{\mathrm{p}}(t_{\mathrm{start}})\Delta\nu + \frac{2R\omega^2}{c}\nu_0\frac{D}{f_{\mathrm{scan}}} < \frac{f_{\mathrm{s}}}{2}. \tag{30}$$

where $c_{\mathrm{p}}(t_{\mathrm{start}}) = \frac{4l_{\mathrm{f}}N\omega}{\nu_0} - \frac{2R\omega^2}{c}t_{\mathrm{start}}$. The spectral resolution $\delta\nu$, which is an inverse of the group delay during the time between t_{start} and t_{end}, may be written as:

$$\begin{aligned}\delta\nu &= \frac{1}{c_{\mathrm{p}}(t_{\mathrm{end}})t_{\mathrm{end}} - c_{\mathrm{p}}(t_{\mathrm{start}})t_{\mathrm{start}}} \\ &= \frac{f_{\mathrm{scan}}}{c_{\mathrm{p}}(t_{\mathrm{start}})D}.\end{aligned} \tag{31}$$

Finally, the trade-off relation is described as:

$$\frac{f_{\mathrm{scan}}}{D}\Delta\nu\frac{1}{\delta\nu} + \frac{8\pi^2 R\nu_0 f_{\mathrm{scan}}}{cP^2}D < \frac{f_{\mathrm{s}}}{2}. \tag{32}$$

Here we express the angular frequency of the scanner as $\omega = \frac{2\pi f_{\mathrm{scan}}}{P}$, where P denotes the number of facets of the scanner. This trade-off relation clearly shows that the nonlinear phase delay broadens the down-converted RF spectrum, leading to slight reduction of the efficiency on the trade-off relation. Note that Supplementary Inequality 32 equals to Supplementary Inequality 24 with $D = 1$ and $R = 0$.

While the Supplementary Inequality 28 gives a constraint on t_{start} caused by the sampling frequency, the geometry of the polygonal scanner also gives another constraint on t_{start}. Since the corners of the polygonal mirror interrupt the scan for one end of the spectrum earlier than for the other end, it gives an additional constraint as:

$$\begin{aligned}t_{\mathrm{start}} &\leq \frac{-\frac{\pi}{P} + \tan^{-1}\left\{\frac{cl_{\mathrm{f}}N}{R}\left(\frac{1}{\nu_0} - \frac{1}{\nu_0 + \Delta\nu}\right)\right\}}{\omega} \\ &\approx -\frac{\pi}{2\,\pi\, f_{\mathrm{scan}}} + \frac{cl_{\mathrm{f}}NP}{R2\,\pi\, f_{\mathrm{scan}}}\left(\frac{1}{\nu_0} - \frac{1}{\nu_0 + \Delta\nu}\right).\end{aligned} \tag{33}$$

In this case, the duty cycle can be described as:

$$D \leq \frac{1}{2} - \frac{Pcl_{\mathrm{f}}N}{2\pi R}\left(\frac{1}{\nu_0} - \frac{1}{\nu_0 + \Delta\nu}\right). \tag{34}$$

Supplementary Note 3: Signal-to-noise ratio.

We evaluate the SNR of single spectra measured by the PC-FTS under the experimental condition of the measurement shown in Fig. 5. Dominant noises are categorized as the detector noise, shot noise and relative intensity noise (RIN) of the light source[1]. In our experiment, an average power irradiated onto the balanced detector is 15 μW for each photodiode, which is limited by the detector nonlinearity. From the noise equivalent power (NEP) of our balanced detector (ca. 4.7 $\text{pW Hz}^{-\frac{1}{2}}$), the SNR dominated by the detector noise is estimated to be 82, while that of the shot noise to be 130. From the measured RIN of our mode-locked laser (-143 dB Hz^{-1}), the SNR dominated by the RIN is estimated to be 255. Therefore, the SNR of our measurement shown in Fig.5 is dominated by the detector noise, and using a detector with a lower NEP could increase the SNR. The overall SNR including all the noises is evaluated to be 66, which is in good agreement with the experimentally measured value of 54. Let us now consider the case using the SLD instead of the mode-locked laser. In this case, relatively larger RIN would dominate the SNR. The measured RIN of our SLD is -124 dB Hz^{-1}, that leads to the SNR of 29 under the experimental condition of the measurement shown in Fig. 5. In addition, the SLD used in our experiment generates a strong ripple noise due to the multiple reflections on the chip surfaces. However, the ripple noise is not a fundamental noise associated to the PC-FTS, which can be eliminated with a proper surface treatment of the chip.

Supplementary Note 4: Spectrum of the fibre laser

The spectrum of the fibre laser used for the measurements of Fig. 4 and 5 is shown in Supplementary Figure 2. The spectrum is measured by an optical spectrum analyzer at a spectral resolution of 0.1 nm that corresponds to 12 – 13 GHz.

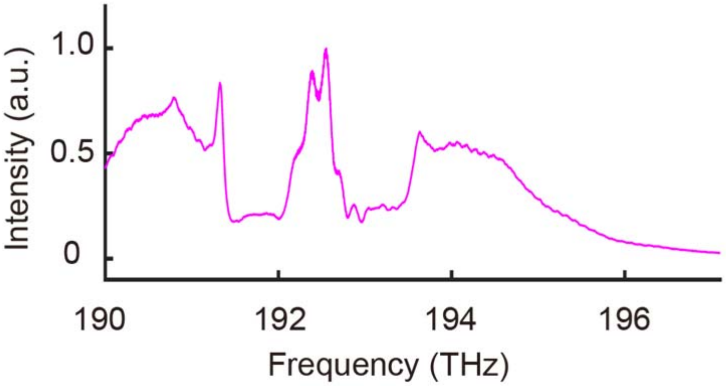

Supplementary Figure 2| Spectrum of the fibre laser.

Supplementary References

1. Newbury, N. R., Coddington, I. & Swann, W. Sensitivity of coherent dual-comb spectroscopy. *Optics Express* **18**, 7929-7945 (2010).
